# Electron Diffusion Length Effect on Direction of Irradiance in Transparent FAPbBr_3_ Perovskite Solar Cells

**DOI:** 10.1021/acs.jpclett.4c02364

**Published:** 2024-09-30

**Authors:** Osbel Almora, Farshad Jafarzadeh, Mohamed Samir, Renán Escalante, Diego Di Girolamo, Jessica Barichello, Francesca Brunetti, Lluis F. Marsal, Fabio Matteocci, Juan Antonio Anta

**Affiliations:** † Department of Electronic, Electric, and Automatic Engineering, Universitat Rovira i Virgili, Tarragona 43007, Spain; ‡ Center for Hybrid and Organic Solar Energy, Department of Electronics Engineering, University of Rome ≪Tor Vergata≫, Via del Politecnico 1, Roma 00133, Italy; § Center for Nanoscience and Sustainable Technologies (CNATS) and Department of Physical, Chemical, and Natural Systems, Universidad Pablo de Olavide, Sevilla 41013, Spain

## Abstract

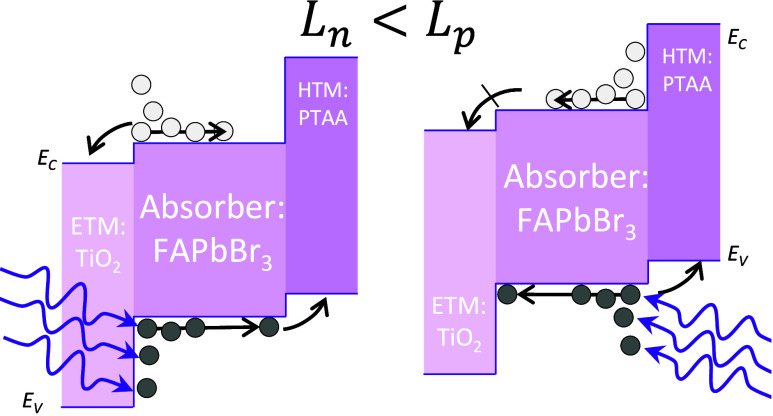

Transparent photovoltaics for building integration represent a promising approach for renewable energy deployment. These devices require transparent electrodes to manage transmittance and to ensure proper cell operation. In this study, transparent FAPbBr_3_-based perovskite solar cells optimized via a passivation treatment were demonstrated with average visible transmittance values above 60% and light utilization efficiencies up to 5.0%. Experiments under varying ultraviolet (UV) irradiance intensities from both front and rear directions revealed performance differences correlated with diffusion-limited transport and open-circuit voltage changes. Combining the UV-radiated experiments and drift-diffusion simulations, an asymmetry between the diffusion lengths of electrons and holes in the perovskite is revealed, with estimated values resulting in less than 50 nm and more than 99 nm, respectively. Our methods not only identify electron–hole diffusion length differences but also introduce a general protocol for characterizing solar cells with transparent electrodes.

Emerging thin film photovoltaics (PVs)^1^ can be designed to use transparent electrodes, and thin absorber layers whose bandgap energy (*E_g_*) can be engineered to absorb a fraction of the incident irradiance and transmit a significant part.^2,3^ This marks a significant difference from the established PV technologies, such as crystalline silicon, where the thick wafers and the *E_g_* invariability hinder the transparency of the devices. Transparent and semitransparent PVs promise opportunities for building integration, such as solar windows and facades,^4−6^ agrivoltaics,^7,8^ and indoor applications.^9,10^ More recently, the advent of transparent luminescent solar concentrators has also been proposed.^11^ However, the device performance and the color tunability of these devices continues to be challenging.

Current state-of-the-art research^1^ shows significant progress among transparent and semitransparent perovskite solar cells (PSCs), which not only shows perspective for building integration^6^ but also paves the way for the development of tandem photovoltaics,^12^ space technology, and the Internet of Things.^13^

Formamidinium lead bromide (FAPbBr_3_) is a perovskite absorber with a large bandgap (*E*_g_ ≈ 2.28 eV) which intrinsically transmits more than half of the visible range spectrum (VIS) and can theoretically reach up to 30% efficiency in tandem configuration with a bottom subcell of *E_g_* ≈ 1.6 eV (e.g., MAPbI_3_). FAPbBr_3_-based PSCs have achieved efficiencies exceeding 10% in opaque devices^14^ and 8% in semitransparent devices.^15^ Specifically, the use of quaternary ammonium halides has demonstrated improvements in performance and long-term stability for these devices.^16,17^ The lead content in FAPbBr_3_ may raise questions on its suitability.^18,19^ However, not only have several encapsulation and recycling strategies already been identified and continue to be under research for mitigating environmental and health impacts^20,21^ but also the perovskite layer in transparent PSCs is typically thinner, which reduces the lead quantity.

The thinner the absorption layer, the higher its transmittance (), leaving aside interference and diffraction phenomena. For transparent PV applications, the focus is set on the visible region of the spectra (VIS), which is characterized by the photopic response of the human eye (*Γ_P_*). Therefore, the relevant parameter to consider is the average visible transmittance:^22^
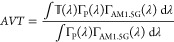
1where *Γ_AM1.5G_*(λ) is the solar spectrum photon flux and the integrals over the wavelength (λ) values between zero and infinity are actually reduced to the visible light range. Ideally, the transparent PV devices would transmit all of the incident photon flux with energies within the visible range and smaller than that of *E*_g_, whereas the remaining photons would be absorbed. In practice, the obtention of convenient transmittance spectra is challenging and the overall absorptance () of the device is reduced as  increases. For an incident photon power density (*P*_in_), the higher the , the smaller the charge carrier generation and thus the electric output power density (*P*_out_), leading to a reduced power conversion efficiency
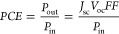
2where *J*_sc_ is the short-circuit density; *V*_oc_, the open-circuit voltage; and *FF*, the fill factor. Therefore, a trade-off is considered between the *AVT* and the *PCE*, which can be linearly balanced with the definition of the light utilization efficiency^23^
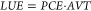
3

Equation 3 quantifies the overall performance of transparent and semitransparent solar cells in a way that, for each *AVT*, the higher the *PCE* the better. This leads back to the fundamental study case in PVs: the reduction of energy losses, either optical or electrical.

Optical losses in transparent devices arise mainly from reflectance () and parasitic  out of the VIS. Notably, the bifacial^24^ functionality of transparent devices not only enhances the versatility of device applications but also indicates the extent to which the optical losses are relevant. Differences between front and rear  and/or  spectra suggest optical issues with transparent electrodes and absorber layers. However, it can be the case where small optical losses cannot explain larger performance differences due to electrical losses when comparing device operation in front and rear irradiance.

The reduction of electrical losses due to nonradiative recombination is arguably the most challenging aspect in the optimization of solar cells. This relates to the difficulty to identify the location of the recombination centers (e.g., trap defects in the bulk or at the interfaces), their nature, and the strategies to mitigate their effects. Most typically, the Shockley–Read–Hall (SRH) recombination is approached in the time domain via the transient spectroscopic experiments for estimating the recombination lifetime for electrons (*τ*_*n*_) and holes (*τ*_*p*_), i.e., the effective time from charge carrier generation until recombination event. However, in the space domain, it is the diffusion length that provides the analogous definition: the average distance a photogenerated charge carrier can diffuse before a recombination event occurs. Importantly, the Einstein’s relation^25^ can be used to relate the electron and holes diffusion coefficients for electrons (*D*_*n*_) and holes (*D*_*p*_). Then, the diffusion lengths for electrons and holes can be approached^26^ to
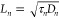
4aand
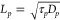
4brespectively, where a direct squared root relation is expected with the lifetimes. Therefore, estimating the diffusion lengths informs both the time and space “life spans” of charge carriers. However, in practice, most of the methods for estimating *L*_*n*,*p*_ follow eq 4 and individually assess the lifetime and the diffusion coefficients throughout the mobility.^27^ This is already controversial, since the techniques employed for each experiment do not necessarily relate to the same variety of phenomena. For instance, bulk or interface contributions can be neglected in one technique or the other, depending on the time scale and/or the location of the perturbation in the sample and the corresponding signal.^28^ Interestingly, Halme et al.^29^ performed intensity modulated photocurrent experiments with dye-sensitized solar cells and found different responses for each direction of illumination; i.e., electrode and electrolyte, demonstrating that an important correlation between the directionality of illumination and diffusion exists when diffusion lengths are on the order of the active layer thickness and the photon absorption penetration lengths are relatively small.

In this work, transparent TiO_2_/FAPbBr_3_/PTAA PSCs with different absorber layer thicknesses and fabrication methods are characterized, and a methodology for assessment of diffusion-limiting transport is introduced. The optoelectronic properties of devices with different thicknesses are established by studying the current density–voltage (*J*–*V*) curve, the external quantum efficiency (*EQE*),^30^ and the  spectra. Moreover, the different sample thicknesses are confirmed via electron scanning microscopy (SEM) images and  spectra. Importantly, the open circuit–voltage, impedance spectroscopy (IS),^31^ and intensity modulated photocurrent (IMPS)^32^ and photovoltage (IMVS)^33^ spectroscopies are explored with front and rear ultraviolet (UV) irradiance. These measurements are correlated with drift-diffusion (DD) simulations and empirical analytical expressions that introduce a method for assessing the differences in perovskite bulk diffusion lengths between electrons and holes. The proposed protocol can be applied to any solar cell with transparent electrodes.

The device performance characterization of the studied samples is presented in Figure 1, and further details on device fabrication and measurement methods can be found in sections S1.1 and S1.2, respectively, in the Supporting Information. Four main sample types were analyzed attending to the thickness, passivation method, and perovskite layer thickness. The reference (ref) samples were exempted of passivation, and the Isoneo samples are those including the interfacial passivation treatment.^13^ Moreover, each type of cell (with/without passivation) was also fabricated with two different concentrations of the precursor solution, 1 and 1.4 M, which resulted in thickness variation of the absorber layer.

**Figure 1:**
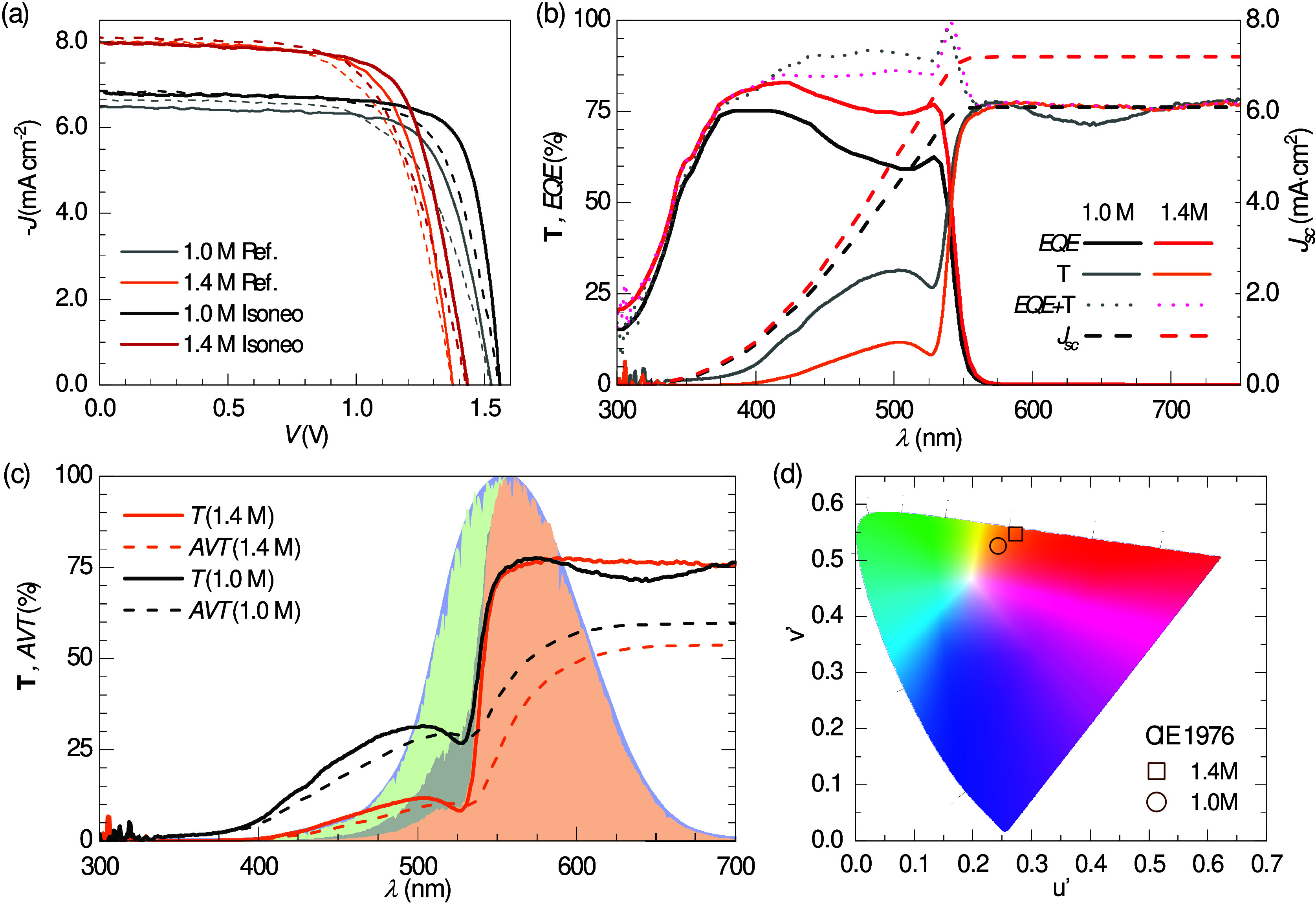
Optoelectronic characterization of the 1 and 1.4 M samples with and without Isoneo passivation in the front direction of illumination. (a) Illustrative *J*–*V* curves under 1 sun illumination; forward and reverse bias scan (100 mV s^–1^) directions are indicated with dashed and solid lines, respectively. The *EQE* spectra for passivated samples are shown in b, and the corresponding transmittance spectra are in b and c. The right axis and dashed lines in (b) indicate the integrated short-circuit current for *Γ*_*AM*1.5*G*_. The dotted lines in b represent the PBCC of eq 5. In c, the dashed lines illustrate the *AVT* integral of eq 1; the blue and green areas correspond to *Γ_P_* and *Γ_P_·Γ_AM1.5G_*, respectively; and the gray and orange areas indicate  for the 1.0 and 1.4 M samples, respectively. The sample color is illustrated in d with the corresponding chromaticity coordinates, and pictures can be found in Figure S3.

The *J*–*V* curves under 1 sun illumination are presented in Figure 1a including bias voltage sweeps in both forward (FW, from short-circuit to open-circuit) and reverse (RV, from open-circuit to short-circuit) directions, with an scan rate of 100 mV·s^–1^, accounting for the hysteresis.^34^ The *PCE* results were similar for the four sample types, around 7%, with slightly higher values for the 1.4 passivated devices (see Table S1). Notably, the *V*_oc_ values are practically independent of the scan rate direction, whereas the *J*_sc_ and the *FF* are affected to some extent by the direction in which the voltage is swept. This agrees with the cumulative statistical data in Figure S1, in the Supporting Information, where the evaluation of the stability of the samples is also illustrated under several test conditions.

Modifying the thickness involves a trade-off between the photocurrent and the photovoltage. Within an optimal range, a thinner sample results in a smaller fraction of absorbed photon flux, leading to a lower generation of charge carriers and, thus, a reduced photocurrent. However, varying the thickness can also alter the morphology and, subsequently, the concentration of recombination sites.

The photocurrent reduction due to the decrease in the thickness of the perovskite layer is also evident in the *EQE* spectra of Figure 1b. The passivated 1.4 M sample shows higher ratios of incident-photons-to-current efficiencies with respect to the 1.0 M sample, resulting in higher values of the integrated *J*_sc_, as shown in the right axis of Figure 1b (dashed lines). Importantly, the *EQE* spectra were further tested with respect to the  spectra in terms of the photon balance consistency check:^22^
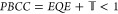
5as presented in Figure 1b with dotted lines. The higher the *EQE* values, the smaller those of the , but in all cases the summation of the spectra does not exceed unity (100%). Only in the absorption threshold does the *PBCC* approach unity, meaning that reflectance () and any further dissipation mechanisms are minimal, if not zero, near the wavelength corresponding to *E*_g_. Notably, the photovoltaic bandgap^30^ of the fabricated FAPbBr_3_-based devices resulted in *E*_g_ = 2.28 eV, as defined by the derivative of the *EQE* spectra^30,35^ in Figure S2, in the Supporting Information.

The integrated *AVT* spectra are included in Figure 1c, as well as the corresponding , the *Γ*_*P*_ spectrum, and the subsequent steps in the integration process of eq 1. First, the total Γ_P_ spectrum is indicated with the blue area, and the green area represents the product *Γ_P_·Γ_AM1.5G_*. Then, the gray and orange areas signify the  products for the 1.0 and 1.4 M passivated samples, resulting in *AVT* values of 59.6% and 53.6%, respectively. These AVT values can be substituted in eq 3 to obtain *LUE* values as high as 5.0%. Furthermore, from the apparent reflectance (see  in Figure 1b) and the clearly transmitted section of the VIS (see Figure 1c), an orangish color for the samples is evident. This is illustrated in the calculation of the uniform chromaticity coordinates (CIE 1976) in Figure 1d and the photographs in Figure S3a. Additionally, the color rendering index (CRI)^22^ resulted in values of 70 and 45.6, for the 1.0 M and the 1.4 M samples, respectively (see details on CRI in section S1.2).

The layer-by-layer structure and optical properties of the samples were also investigated, as summarized in Figure S3b–f, in the Supporting Information. The SEM cross-section images in combination with the analyses of , , , and the effective uniform absorption coefficient (*α*) spectra of the 1.0 and 1.4 M samples suggest effective thicknesses (*L*) of approximately 150 and 255 nm, respectively. Importantly, the estimation of α follows the Beer–Lambert (B-L) law,^36^ which gives the position *x*-dependent monochromatic photon flux
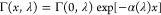
6for an incident monochromatic flux *Γ*(0, λ) of photons just inside the surface of the material (i.e., after accounting for reflection), with wavelength *λ*. Moreover, the B-L law introduces the useful concept of photon penetration length or effective charge carrier generation region of width *w* = 1/α. Particularly, the studied FAPbBr_3_ layers showed *w* < 100 nm for λ < 400 nm (see Figure S3f). This suggests that UV irradiance would be totally absorbed in the perovskite layers within regions smaller than their thicknesses (*w_UV_* < *L*), in agreement with the negligible  spectra for the 1.4 M sample in Figure 1c,d.

Some performance differences between the samples can also be identified from IS measurements in quasi-open-circuits under different white LED illumination intensities. The experimental spectra were fitted to the equivalent circuit in Figure S4, and the resulting analyses are summarized for the 1.0 and 1.4 M samples in Figures S5 and Figure S6, in the Supporting Information, respectively. In general, higher resistance (*R*) can be identified for the passivation-treated devices with respect to the nonpassivated ones. This suggests an increase in the recombination resistance for the Isoneo samples, whose nonradiative recombination may have been reduced. The capacitance (*C*) showed a drastic decrease for the passivated sample with respect to the reference among the 1.0 M devices but nearly unchanged spectra for the 1.4 M samples. These features may be related to the competition between geometric capacitances from the perovskite layer and those of the selective contacts and interfaces.

The passivated samples were further studied by means of alternating-current mode (AC) techniques including IS, IMPS, and IMVS spectra in (quasi-) open-circuit conditions under different irradiance intensities. However, in order to probe the impact of the recombination and the diffusion lengths, in this case, the illumination was provided by a 365 nm monochromatic UV-LED (PAIOS Fluxim), and a comparison was made between the front and rear directions of irradiance, herein defined as those where the photon flux is interacting with the glass/FTO/TiO_2_ and ITO/PTAA layers before the perovskite, respectively. This front/rear distinction is graphically illustrated in the simulated charge carrier generation rate (*G*) profile in Figure 2a (SETFOS Fluxim, see section S2 in the Supporting Information) and the layer-by-layer inset diagram in Figure 2b.

**Figure 2:**
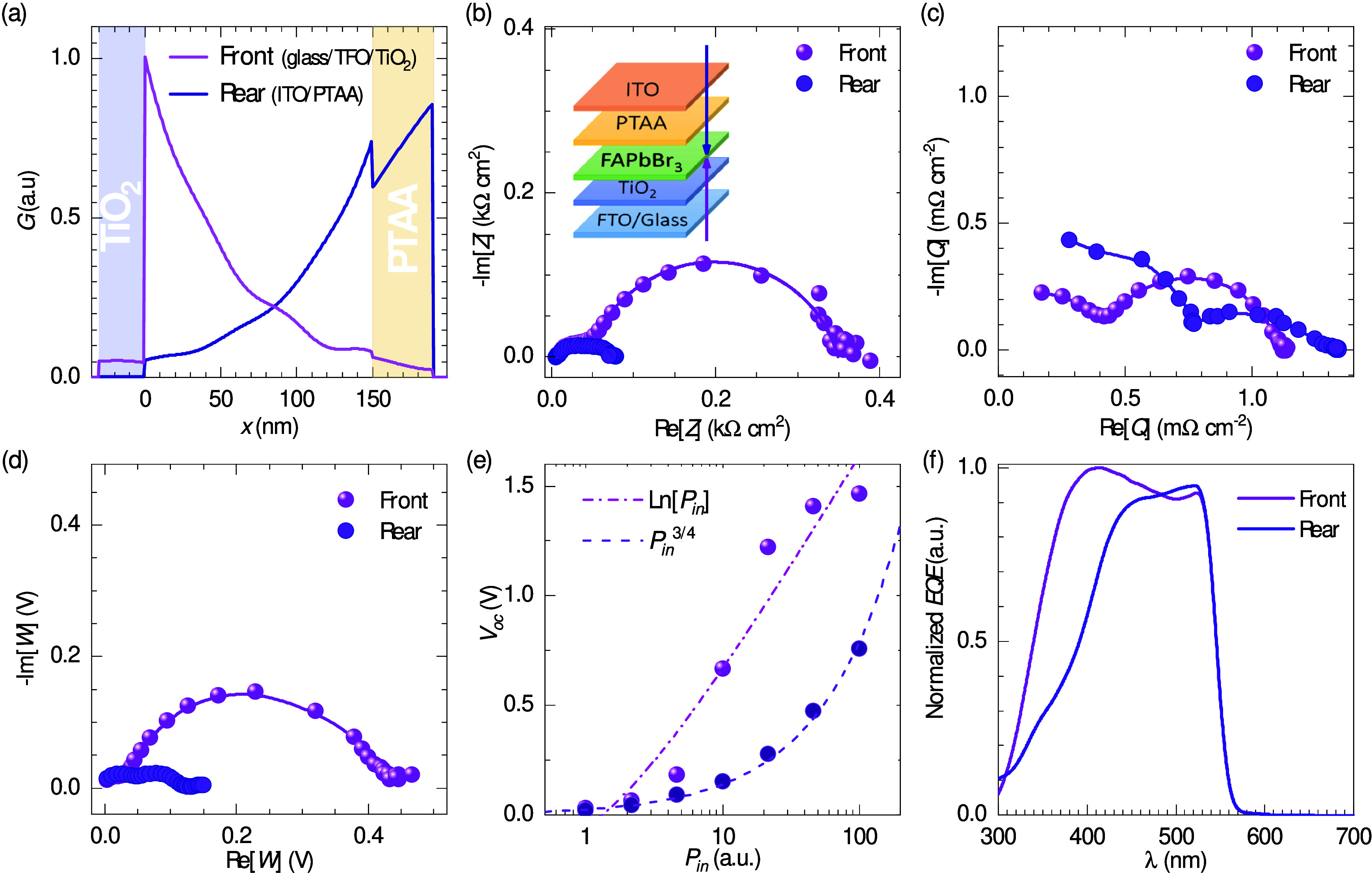
Front and rear directions of UV irradiance, as indicated: (a) simulated charge carrier generation rate for incident 365 nm photons into the 1.0 M sample; illustrative experiments with Isoneo 1.4 M samples including (b) IS, (c) IMPS, and (d) IMVS spectra under (quasi-) open-circuit conditions for the highest UV irradiance intensities; (e) DC open-circuit voltage as a function of normalized incident power densities; and (f) *EQE* spectra. The inset in b illustrates the layer-by-layer structure of the samples.

The IS characterization of a passivated (thinner) 1.0 M sample in quasi-open-circuit under different UV irradiance intensities shows similar spectra for both front and rear directions of radiance, as presented in Figure S7, in the Supporting Information. This agrees with the *G* profile in Figure 2a, since the perovskite layer thickness (∼150 nm) is close to the photon penetration length. Moreover, the equivalent circuit modeling (Figure S4) was employed to obtain the *C*, *R*, and *τ* summarized in Figure S8a–c, respectively, in the Supporting Information. These parameters reproduce a similar behavior with a slight increase in *C* and *R* (and subsequently *τ*) for the front illumination. Furthermore, the *V_oc_* values as a function of the incident illumination intensities, and the *EQE* spectra are also presented in Figure S8d,e, confirming the effective similarity between the electrical response of the sample in both directions of illumination for the (thinner) 1.0 M sample.

A thicker (1.4 M) passivated sample was also explored via IS in quasi-open-circuit conditions under different UV illumination intensities, as shown in Figure S9 and summarized in Figure 2b–d. However, unlike the thinner sample, in this case clear differences were obtained when comparing front and rear irradiance directions. For instance, the impedance (*Z*) Nyquist plot from IS measurements at the highest UV direct-current mode (DC) irradiance intensity in Figure 2b illustrates a significant increase of resistance (*R*) for the front illumination with respect to that of the rear irradiance. This difference is significantly dependent on the irradiance intensity, as shown in Figure S10.

The photocurrent and photovoltage responsivities, *Q* and *W*, from IMPS and IMVS are presented in Figure 2c,d, respectively, and for analogue DC conditions to those of the IS in Figure 2b. Two characteristic semicircles are evident in all spectra in Nyquist plot representation, but it is the higher photovoltage *W* response in the front direction of irradiance, with respect to the rear one, that marks the main contribution to the resistance (*Z* = *W*/*Q*).^33^ In addition, a clear resemblance is found between the IMVS spectra and the corresponding spectra from the IS measurements. This not only validates the consistency of the measurement protocol^32^ but also suggests the recombination nature of the resistance in quasi-open-circuit conditions.^37^

The DC measurements of *V*_oc_ as a function of the incident UV irradiance intensity (Figure 2e) reveal higher photovoltage values when irradiated from the front compared with the rear. The higher the intensity of irradiance, the higher the difference in *V_oc_* values, although a saturation of the trend is also observed for the highest UV intensities. While the front *V_oc_* shows the typical logarithm increase with the *P*_in_, the rear one apparently follows a 3/4-power law. This suggests potential UV-activated phase transitions modifying transport parameters with rear irradiance, whose full comprehension is beyond the scope of this manuscript. More importantly, the consistent observation of significantly higher *V_oc_* values in the front compared to the rear direction of irradiance cannot be solely related to the higher charge carrier generation (Figure 2a). Therefore, higher recombination lifetimes appear for the front compared to the rear direction, accounting for the results from both the AC and DC experiments in open-circuit conditions (Figure 2b–e).

The DC *EQE* spectra in short-circuit conditions are presented in Figure 2f, comparing the effect of different directions of irradiance. Unlike the thinner 1.0 M sample (see Figure S8), the 1.4 M device shows a clear reduction in the spectrum from the rear when compared to front as the wavelength approaches the UV range, for *λ* < 400 nm. Since short-circuit conditions favor transport over recombination, the higher photocurrent responsivity in the front direction can be associated with transport differences in diffusion lengths rather than solely recombination lifetimes, as one may conclude from the experiments conducted under open-circuit conditions (Figure 2b–e).

The understanding of our experiments was contrasted with DD simulations (see section S2 in the Supporting Information). First, the increase of *J*_sc_ and decrease of *V*_oc_ in the *J*–*V* curves in Figure 1a, as the thickness *L* of the perovskite layer increased from the 1.0 M to the 1.4 M samples, is qualitatively reproduced in Figure S11. This served as a calibration for the simulation parameters in Table S3 and also indicated a decrease of bulk recombination lifetime for the thicker perovskite layer compared to the thinner one, possibly due to a higher concentration of bulk recombining traps.

Second, several simulations were conducted to reproduce a difference in electrical response between front and rear directions of irradiance, given the absorption coefficient of a 255-nm-thick FAPbBr_3_ layer (see Figure S3). This included three main hypotheses for the imbalance of the electrical response: (i) changes in recombination velocities at the interfaces, (ii) ion-mediated phenomena, and (iii) differences in *L*_*n*_ and *L*_*p*_ in the bulk of the perovskite. None of the explored ranges of simulation parameters within the framework of SETFOS-Fluxim reported significant electrical response differences between front and rear directions of irradiance for hypotheses i and ii. In contrast, it was only with the assumption of *L*_*n*_*≪ L*_*p*_ that the current, recombination resistance, and photovoltage resulted in higher values in the front compared to the rear direction of UV irradiance. For instance, Figure S12 illustrates the qualitative reproduction of the experimental trends for the IS spectra and *V_oc_–P_in_* in the front and rear directions of UV irradiance. Particularly, our best qualitative agreement between experiments and simulations was obtained for the diffusion lengths of electrons and holes in the ranges of *L*_*n*_ < 50 nm and *L*_*p*_ > 99 nm, respectively.

Notably, it could be argued that the directional effect could be due to the parasitic absorption of the contacts, e.g., the PTAA (see Figure 2a). However, the stronger decrease of *V_oc_* with rear irradiance from the thicker perovskite layer, with respect to that of the thinner one, suggests that it is the asymmetry of the diffusion lengths within the perovskite layer that determines the *V_oc_* decrease with the irradiance direction, instead of the PTAA. To confirm this hypothesis, we performed additional DD simulations with varying PTAA thicknesses, showing that for thicknesses > 100 nm, the *V_oc_* drop due to PTAA absorption would limit the perovskite-focused analysis (see Figure S11b in the Supporting Information). In contrast, for PTAA thicknesses < 50 nm, it is safe to assume that most of the contribution to the irradiance direction-dependent response is due to the perovskite.

Our experimental observations and numerical simulations suggest that a systematic increase (decrease) in photocurrent and photovoltage occurs for front compared to rear directions of monochromatic irradiance when *L*_*n*_ ≪ *L*_*p*_ (*L*_*n*_ ≫ *L*_*p*_) in the bulk of the absorber layers whose *w* ≪ *L* in both directions, in p-i-n devices with transparent electrodes. In general, a dependence on the direction of illumination will show up whenever there is an asymmetry in the respective diffusion lengths of electrons and holes (see below). This is typically the case for transparent solar cells under UV irradiance. Nevertheless, it is strongly recommended to validate these analyses with experiments comparing samples with thinner and thicker absorber layers with the same transport layers since the UV absorption of some of the transport materials (e.g., PTAA) could also contribute to the direction-dependent response.

The above reasoning is better explained with the aid of the energy diagrams in Figure 3a,b that summarize the differences between the front and rear directions of irradiance, respectively, considering the main transport and recombination mechanisms. Regardless of whether the incident photon flux accesses the device through the electron or hole transport material, ETM and HTM, respectively, four main processes take place: (1) photon absorption by electrons with energies smaller than that of the conduction band maximum (*E*_*V*_); (2) charge carrier generation exciting an electron to energies above the minimum *E*_*C*_ and leaving a hole in the valence band; (3) drift and diffusion of electrons and holes toward the ETM and the HTM, respectively; and (4) charge carrier recombination. These processes are well-known and occur in p-i-n devices as long as there is a built-in field and the diffusion lengths of electrons and holes are different and complementary in the selective contacts. As a result, the drift-diffusion current of electrons and holes, *J*_*n*,*DD*_ and *J*_*p*,*DD*_, in the direction of the built-in field (*ξ_bi_*) occur as the charge carriers are extracted toward the ETM and HTM, respectively.

**Figure 3:**
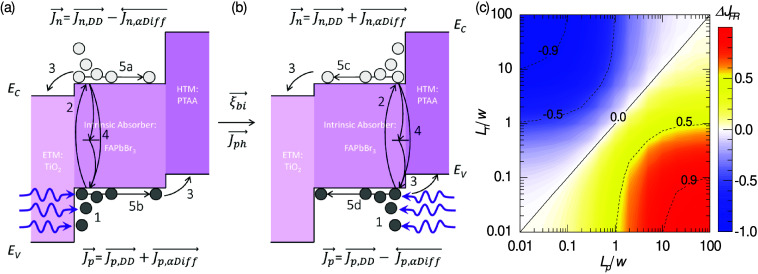
Illustrative energy diagram for a TiO_2_/FAPbBr_3_/PTAA solar cell with transparent electrodes under monochromatic UV irradiation from the (a) front (ETM: TiO_2_) and (b) rear (HTL: PTAA) electrodes and (c) normalized direction current difference of eq 9. Main processes include (1) photon absorption, (2) charge carrier generation, (3) charge extraction through drift and diffusion toward the selective contacts, and (4) recombination, in the diagram. When photon penetration length is smaller than the thickness of the absorber layer (*w* < *L*), B-L absorption-related (5) diffusion of charge carriers occur. In (a) front irradiation, (5a) electrons and (5b) holes diffuse toward the HTL, whereas for (b) rear irradiance, (5c) electrons and (5d) holes diffuse toward the ETL. In between the two diagrams, the direction of built-in field and photocurrent is indicated.

The drift or diffusion contributions to *J*_*n*,*DD*_ or *J*_*p*,*DD*_, and their proportion to the total current *J* = *J*_*n*,*DD*_+ *J*_*p*,*DD*_, depend on the device architecture and properties, the bias, and incident irradiance. For example, in short-circuit conditions, *J* = *J*_ph_ typically has a main contribution from drift current.^38^ Importantly, DD current components can also occur in the direction opposite to that of the *ξ_bi_*, and *J*_ph_, which would reduce the performance of the devices (e.g., due to field ion screening).^39−42^ However, in the following, we assume these contributions to be independent of the direction of irradiance, meaning that they are already accounted for in *J*_*n*,*DD*_ and *J*_*p*,*DD*_, which are also fundamentally independent of the direction of irradiance.

The introduction of B-L absorption profiles with *w* ≪ *L* in Figure 3 illustrates how the photogeneration profile cannot be approximated to a homogeneous distribution and diffusion current of electrons (*J*_*n*,αDiff_) and holes (*J*_*p*,αDiff_) takes place. The absolute values of *J*_*n*,αDiff_ and *J*_*n*,αDiff_ are assumed to be approximately the same, regardless of the direction of irradiance of monochromatic photons with absorption coefficient α. However, for the front direction of irradiance (Figure 3a), electrons (5a) and holes (5b) diffuse toward the HTM, in the directions against and in favor to the current, respectively. In contrast, in the rear direction of irradiance (Figure 3b), electrons (5c) and holes (5b) diffuse toward the ETM, in the directions in favor and against the current, respectively. Therefore, the current difference between front and rear directions of irradiance can be approximated to
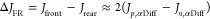
7An exact solution for expressing eq 7 in terms of the diffusion lengths and other fundamental transport properties may require numerical methods. However, considering (i) an approximation to the solution of the continuity equation for a diffusion process with a monochromatic B-L generation rate at the edge of the depletion region^43^ and (ii) the nonhomogeneous photogeneration (*w* < *L*), one can estimate:
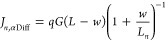
8aand
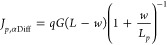
8bThen, substituting eq 8 into eq 7, it leads to the empirical photocurrent direction difference
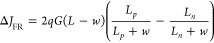
9From eq 9 we can conclude that (i) no current difference depending on the direction of irradiance is obtained for quasi-homogeneous photogeneration (*w* > *L* ⇒ Δ*J*_FR_ = 0) or (ii) identical diffusion lengths (*L*_*n*_ = *L*_*p*_ ⇒ Δ*J*_FR_ = 0) and (iii) that the sign of the direction current difference depends on the diffusion lifetimes: *L*_*p*__>_^<^*L*_*n*_ ⇒ Δ*J*_*FR*__>_^<^ 0. Importantly, not only is *L*_*p*_ ≠ *L*_*n*_ required for obtaining a measurable value Δ*J*_FR_ ≠ 0, but also there is a close relation with *w*. Expression 9 can be further extended to the open-circuit condition, resulting in an empirical photovoltage direction difference
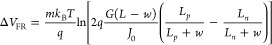
10where *m* is the ideality factor, *k*_B_*T*/*q* is the thermal voltage, and *J*_0_ is the dark saturation current of the sample.

The normalized values of eq 9 as a function of the ratios *L*_*p*_/*w* and *L*_*n*_/*w* are presented in Figure 3c, which clearly equals zero in the diagonal, where *L*_*n*_ = *L*_*p*_. However, for diffusion lengths with values similar to the photon penetration depth (*L_n_*, *L_p_* ∼ *w*), smaller differences between *L*_*n*_ and *L*_*p*_ produce higher current direction differences (smaller distance between negative-blue and positive-red regions in Figure 3c). In contrast, for diffusion lengths with values significantly smaller or higher than the photon penetration depth (*L*_*n*_, *L*_*p*_ ≪ *w* or *L*_*n*_, *L*_*p*_ ≫ *w*), it takes higher differences between *L*_*n*_ and *L*_*p*_ to produce significant current direction differences (white-near-zero areas in Figure 3c).

In summary, this study investigated the impact of the difference between electron and hole diffusion lengths on the direction of UV irradiance in transparent FAPbBr_3_-based PSCs. The devices were fabricated and optimized by including an Isoneo passivation treatment, which not only increased the device *PCE* but also allowed *AVT* values to be as high as 60% for *LUE* values above 5%. The superior performance of the passivated devices was confirmed through various characterization techniques, suggesting a reduction in the nonradiative recombination compared to the reference nonpassivated samples.

The thickness, illumination spectra, and direction of irradiance were studied and correlated with fundamental electron–hole transport properties. The IS analysis of samples thicker than 150 nm under 365 nm UV irradiance from both the front and rear directions demonstrated significant differences in open-circuit and short-circuit conditions, whereas experiments under white LED illumination did not show such trends. Our numerical simulations and the experimental results suggest that the electrons and holes diffusion lengths in the studied FAPbBr_3_ transparent samples are smaller than 50 nm and greater than 99 nm, respectively.

A discussion on diffusion transport for Beer–Lambert monochromatic absorption was provided, along with empirical approximations for the photocurrent and photovoltage differences between measurements taken from the front and rear directions of irradiance. The characterization protocol introduced here facilitates the identification of the limiting diffusion length requiring further optimization, not only in semitransparent PSCs but also for solar cells with semitransparent electrodes in general. Moreover, the estimation of *L*_*n*_, *L*_*p*_, and their difference, could also be extended to samples with several other absorber layer thicknesses, and extra measurement protocols could be used for contrasting and validating the results.

## Supporting Information Available

Additional experimental details, materials, and methods, including photographs of the samples; performance statistics and stability tests; absorption, transmittance and impedance spectra for different irradiance intensities, spectra and directions; drift-diffusion simulation parameters (PDF)
