# Supplementary material for: Electron Diffusion Length Effect on Direction of Irradiance in Transparent FAPbBr3 Perovskite Solar Cells
Source: J Phys Chem Lett. 2024 Sep 30;15(40):10153–61. doi: 10.1021/acs.jpclett.4c02364 (PMC11634021; doi:10.1021/acs.jpclett.4c02364)
Supplement: Supplementary file 1 — jz4c02364_si_001.pdf [file jz4c02364_si_001.pdf]

Supporting information:

Electron Diffusion Length Effect on Direction of Irradiance in Transparent $FAPbBr_3$ Perovskite Solar Cells

Osbel Almora[1,]*, Farshad Jafarzadeh,[2] Mohamed Samir,[1] Renán Escalante,[3] Diego Di Girolamo[2], Jessica Barichello[2], Francesca Brunetti[2], Lluis F. Marsal,[2] Fabio Matteocci[1,]*, and Juan Antonio Anta,[3,]*

[1] *Department of Electronic, Electric and Automatic Engineering, Universitat Rovira i Virgili, 43007 Tarragona, Spain*

[2] *Center for Hybrid and Organic Solar Energy, Department of Electronics Engineering University of Rome «Tor Vergata», Rome, Italy*

[3] *Department of Physical, Chemical and Natural Systems, Universidad Pablo de Olavide University, Sevilla 41013, Spain*

* corresponding authors: osbel.almora@urv.cat; fabio.matteocci@uniroma2.it, jaantmon@upo.es

Keywords: Perovskite solar cells; transparent photovoltaics; impedance spectroscopy; charge carrier recombination

S1. Experimental

S1.1. Materials and fabrication methods

Semi-transparent Perovskite solar cells (ST-PSCs) were fabricated with the device structure of Glass/FTO/c-TiO_2/SnO_2/$FAPbBr_3$/PTAA/ITO. Initially, Glass/FTO substrates (TEC15 Pilkington, 15 Ω/square) were cut into 2.5 × 2.5 cm^2 size and patterned using a nanosecond Nd:YVO_4 laser (λ = 355 nm, 15 ns, pulsed at 80 kHz) with a fluence per pulse of 715 mJ cm^{-2}. Substrates underwent a 10-minute cleaning process in an ultrasonic bath using detergent, deionized water, and 2-propanol sequentially.

A compact TiO_2 layer (40 nm thickness) was deposited through spray pyrolysis at 460°C using a precursor of titanium diisopropoxide bis(acetylacetonate):acetylacetone:ethanol (in a 3:2:45 volume ratio). Subsequently, a 1:20 v/v aqueous solution of SnO_2 nanoparticles (Alfa Aesar) in deionized water was spin-coated onto the TiO_2 electrodes at 4000 rpm for 20 s, followed by annealing at 120°C for 15 minutes.

In a nitrogen-filled glovebox, the $FAPbBr_3$ perovskite precursor solution was prepared at two different concentrations of 1M or 1.4M. $PbBr_2$ (99.99% purity, Tokyo Chemical Industry) and FABr (99.99% purity, Greatcell Solar Materials) in Dimethyl sulfoxide with desired concentration dissolved in DMSO (anhydrous, ≥99.9% Sigma-Aldrich). The solutions were then spin-coated onto substrates at 4000 rpm for 20 seconds. Ethyl acetate was introduced as an antisolvent in the final 10 seconds, and substrates were annealed at 80°C for 10 minutes. For cells with interfacial passivation treatment, a solution with a 1 mg/ml concentration of Neo-Pentylammonium chloride and iso-Pentylammonium chloride (Greatcell Solar Materials), prepared in a 1:1 v/v ratio in 2-propanol, was spin-coated at 4000 rpm for 20 s, according to our previous work.[1]

Poly[bis(4-phenyl)(2,4,6- trimethylphenyl) amine] (PTAA), 10 kDa by Solaris Chem was dissolved in toluene (Sigma-Aldrich) at a concentration of 10 mg/ml, followed by the addition of tert-butylpyridine (TBP), 10µl/ml and Li bis(trifuoromethanesulfonyl)imide (5µl/ml, stock solution: 170mg/ml in acetonitrile). The PTAA film was deposited at 4000 rpm for 20 seconds.

Low-temperature ITO deposition was conducted using an industrial in-line magnetron sputtering system (KENOSISTEC S.R.L., KS 400 In-Line) at 1.1 × 10^{-3} mBar and 90W

RF power. During ITO deposition, inert Ar gas (40sccm) was purged in the chamber to activate the Ar^+ plasma. The sample holder was moved below the ITO cathode at a speed of 120 cm/min for 200 cycles to achieve a thickness of 150 nm.

S1.2. Characterization

All solar cells were measured with the active-area of 0.3 cm^2 using a Class A solar simulator (ABET Sun 2000) under AM1.5G conditions calibrated with a reference silicon solar cell (RR226-O, RERA Solutions). The $J - V$ measurements under forward and reverse scan directions were done using Arkeo platform (Cicci Research s.r.l.), resulting in the data of Figure 2a (in the main manuscript), **Figure S1** and **Table S1**. The EQE spectra were measured using a measurement system from Arkeo–Cicci research s.r.l. in short-circuit with/without background bias illumination, at an alternating current (AC) chopper frequency (f) of 16.6 Hz. Another Lasing IPCE-DC model equipment with the series number LS1109-232 was employed for the experiments in rear and front irradiance. The output EQE spectra are shown in Figure 2b,c and **Figure S2**. The ISOS-D2[2] stability test involved storing samples in an oven at 85°C in ambient air and measuring them under AM1.5G light conditions. The light soaking test is conducted using an ARKEO light soaker (VIS version) equipped with LED light sources calibrated to 0.7 sun intensity.

A summary of layer thicknesses is shown in **Table S2**, in the supporting information, and the corresponding scanning electron microscopy (SEM) cross section images, captured using a TESCAN MIRA microscope, are in Figure 3a,b (main manuscript). Further device and layer-by-layer optical characterizations were performed with a spectrometer (Shimadzu UV- 2550), resulting in the data in Figure 3c,d and **Figure S3**.

The IS[3] was measured with an Autolab PGSTAT302N potentiostat including a FRA32M unit and a kit Autolab Optical Bench from MetroOhm for the white illumination and with a PAIOS station from Fluxim for the UV illumination (364 nm). The utilized white light emitting diode (LED) was a CREE XM-L3 U4 on Star PCB XMLDWT-U40E1. In all the cases the IS spectra were measured in quasi-open-circuit conditions[4] by setting different steady-state illumination intensities, then stabilizing the V_{oc} before applying the corresponding forward bias that cancels the direct current (DC) operation. Upon these DC conditions the 15 mV perturbation was applied for measuring the IS as a function of the V_{oc} for each illumination intensity.

Drift-diffusion numerical simulations of the *J-V* absorption profile and IS spectra were conducted with Setfos Fluxim,[5] including the simulation parameters in **Table S3**. Furthermore, analytical simulations of the IS spectra were fitted to the equivalent circuit (EC) model of **Figure S4**.

The Color Rendering Index (CRI) is widely used in the lighting and display industries to measure how closely an artificial light source mimics a blackbody radiator spectrum at a specific color temperature. CRI ranges from 0 to 100, with values above 90 considered excellent for lighting. The CRI was calculated according to the work of Yang et al.[6] that also includes a data analysis Excel sheet that extracts CRI, provided a transmittance spectrum.

The CIE 1931 XYZ color matching functions (integrated with a source spectra AM1.5G or AM1.5G·T) yield tristimulus values (X, Y, Z), which are then converted to (x, y) coordinates in CIE 1931 color space. For AM1.5G, the (x, y) coordinates are (0.3322, 0.3439) with a CCT of 5513 K. In CRI calculations, these tristimulus values are converted to (u, v) coordinates in CIE 1960 uniform color space (CIELUV). As selective harvesting cut-off shifts, the (u, v) coordinates in CIELUV approach the AM 1.5G reference point, enhancing transparency and minimizing tinting.

Color is defined by chromaticity (u, v) and luminous intensity (L). A color sample will show differences in chromaticity (Δu^* and Δv^*) and lightness (ΔL) when illuminated by a reference or transmitted source. The chromaticity difference between a transmitted source and AM1.5G is illustrated by the geometric distance between their points in the chromaticity coordinate system. The CRI is calculated by averaging the chromaticity and lightness differences for eight standard test-color samples.

In CIELAB, (L^*, a^*, b^*) values are derived from the tristimulus values of both the reference and transmitted sources. The reference source, referred to as the "white point," should be the AM 1.5G spectrum. The calculation of L^* is consistent for both CRI and CIELAB.

$$CRI = 8\sum_{i=1}^{8}\left[100 - 4.6\sqrt{(\Delta u_i)^2 + (\Delta v_i)^2 + (\Delta L_i^*)^2}\right]$$

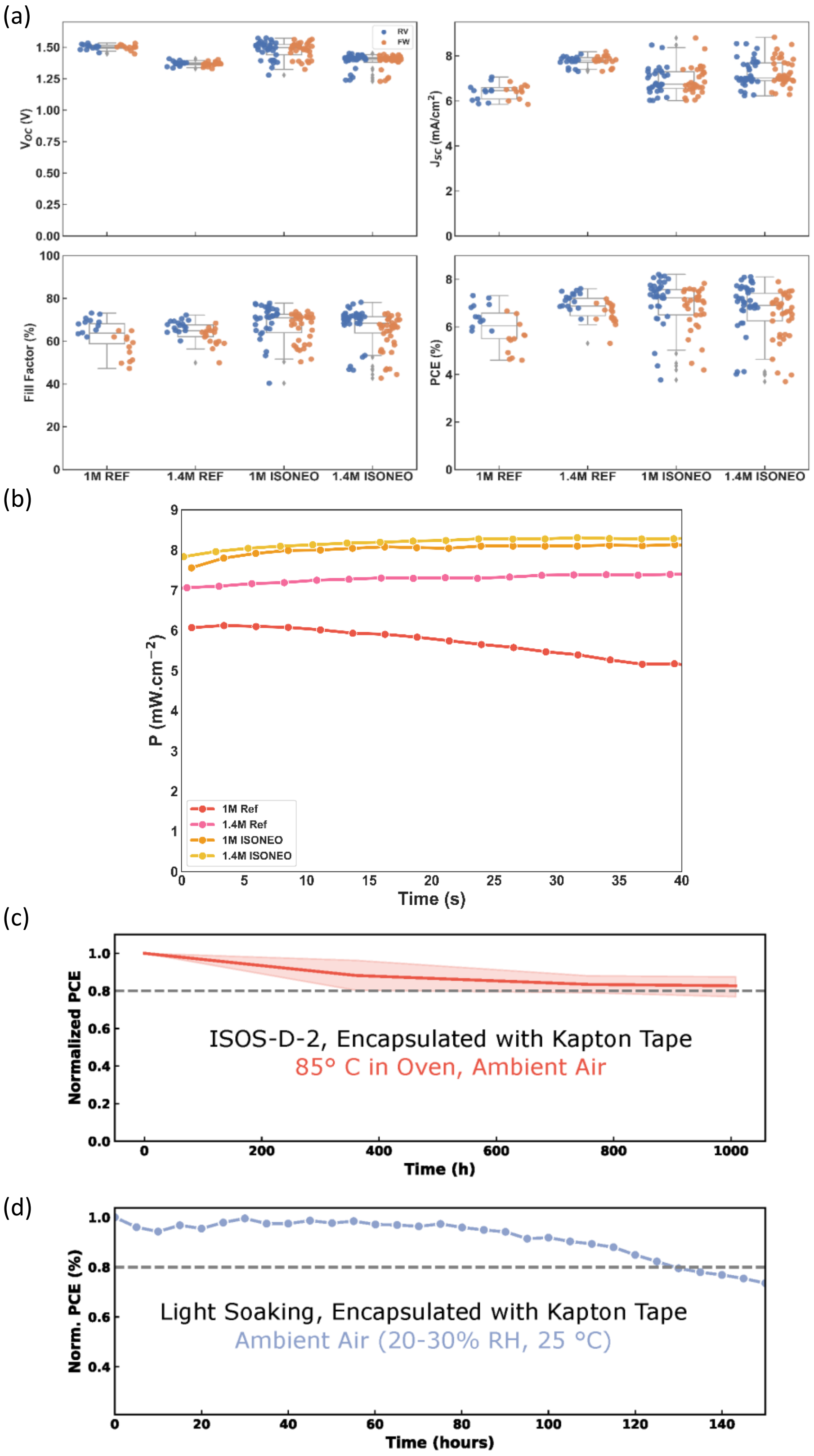

Figure S1. (a) Statistical summary of performance parameters under standard 1 sun AM1.5G illumination intensity for the studied samples, as indicated. Top panel includes (left) open-circuit voltage (V_{oc}) and (right) short-circuit current density (J_{sc}), whereas the bottom panel shows (left) the fill factor (*FF*) and (right) the power conversion efficiency (*PCE*). (b) MPP tracking under standard 1 sun AM1.5G illumination intensity for the studied samples, as indicated. (c) Thermal stability test (ISOS-D-2) measured for four devices with 1M $FAPbBr_3$ and ISONEO treatment (on average 82% of initial *PCE* after 700 h). (d) Light soaking stability test measured for a device with 1M $FAPbBr_3$ and ISONEO treatment (retained 80% of initial *PCE* after 130 h).

Table S1. Performance parameters from J-V curves in **Figure 2** in the main manuscript for the best cells and corresponding average visible transmittance (AVT) and light utilization efficiency (LUE). The "ave" subscript indicate average between the two scan rate directions.

Group	Scan Direction	V_{OC} (V)	J_{SC} (mA.cm^{-2})	FF (%)	PCE (%)	PCE_{ave} (%)	AVT (%)	LUE (%)
Ref- 1M	FW	1.52	6.64	64.92	6.56	6.89		
	RV	1.52	6.47	73.15	7.22			
ISONEO-1M	FW	1.55	6.84	71.21	7.57	7.89	59.6	4.52
	RV	1.56	6.78	77.56	8.21			5.00
Ref- 1.4 M	FW	1.37	8.02	63.92	7.03	7.32		
	RV	1.37	7.97	69.34	7.61			
ISONEO-1.4M	FW	1.43	8.09	63.60	7.35	7.73	53.6	3.94
	RV	1.43	7.96	71.03	8.11			4.35

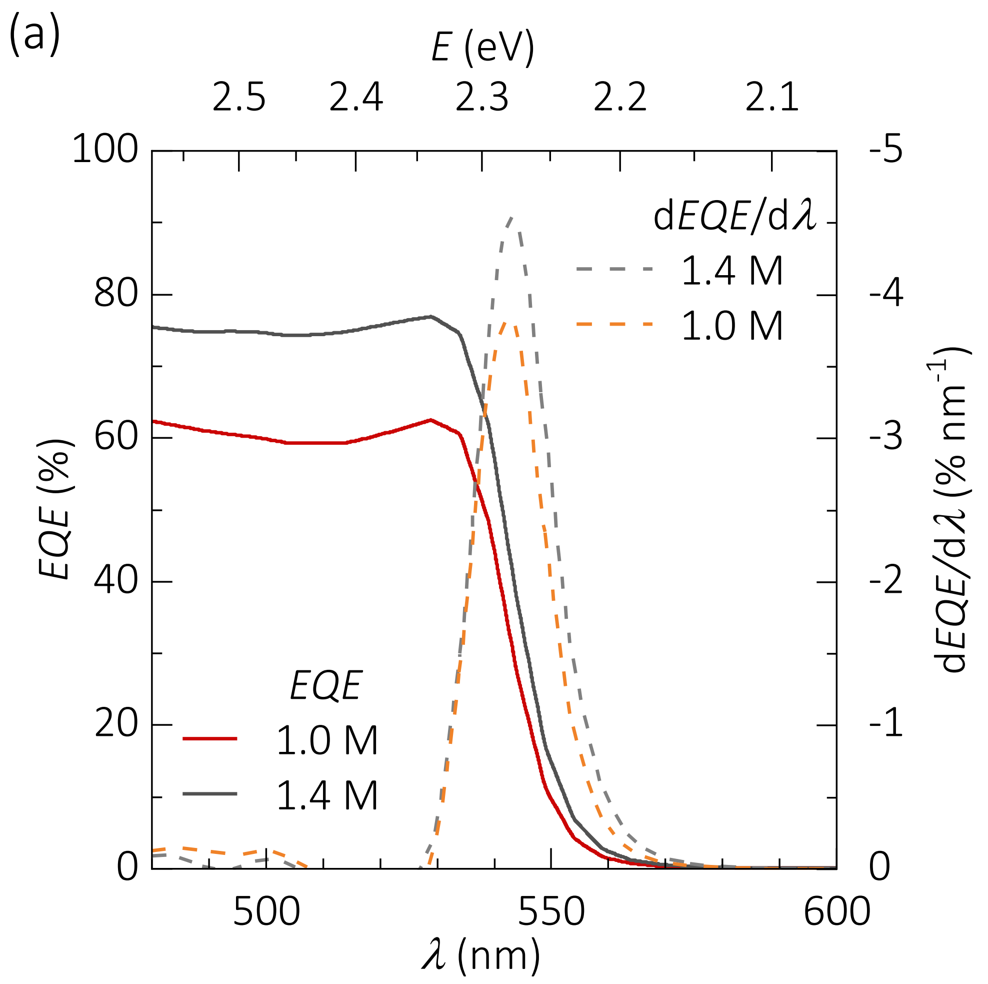

Figure S2. Evaluation of the photovoltaic bandgap[7] of the $FAPbBr_3$-based devices with different perovskite thicknesses: 1.M (150 nm) and 1.4M (255 nm).

Table S2. Material layer-by-layer thicknesses for the studied samples

Layer	Thickness (nm)
TiO_2	30±10
$FAPbBr_3$ 1M	150±18
$FAPbBr_3$ 1.4M	255±14
PTAA	40±10
ITO	100±10

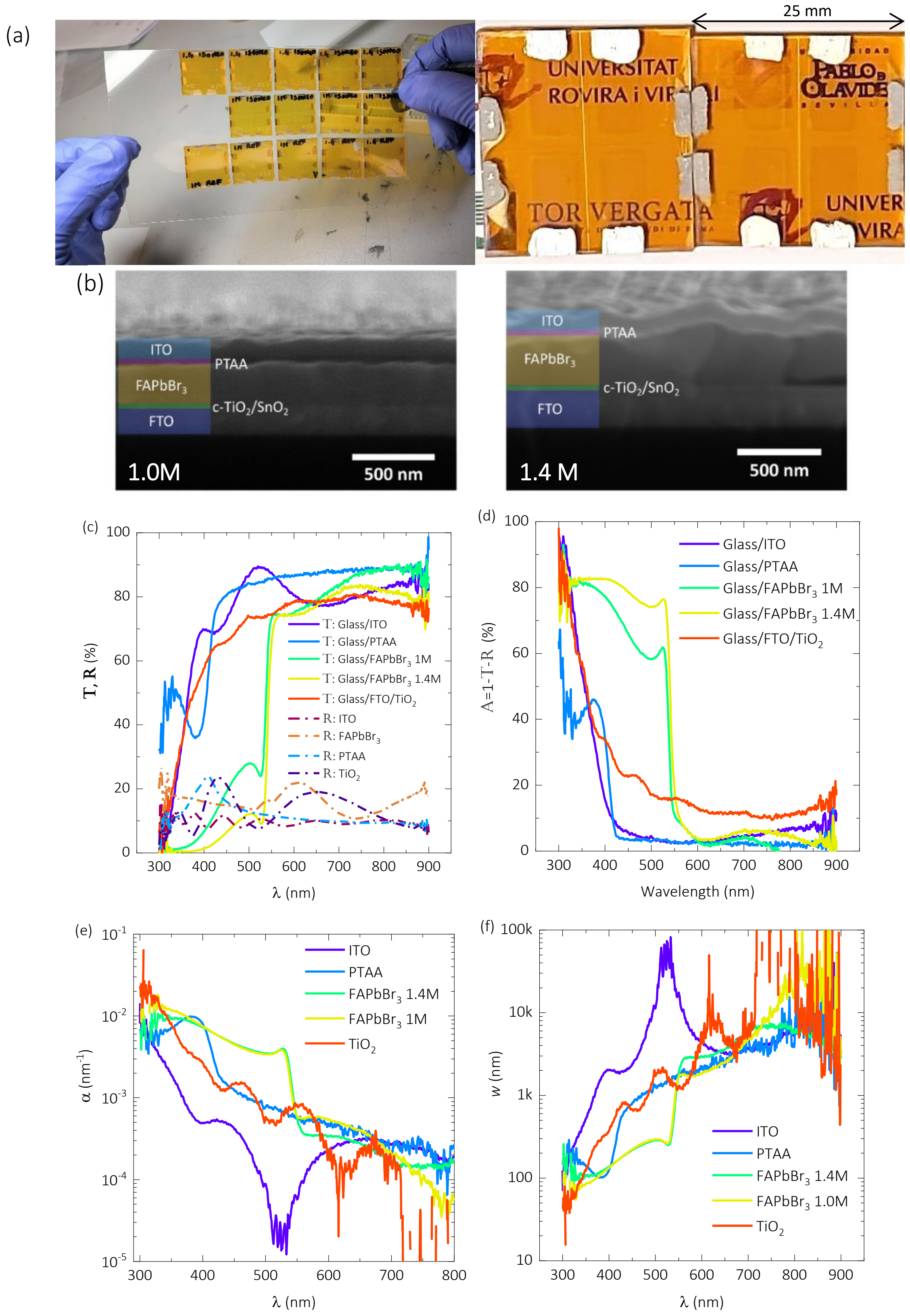

Figure S3. Optical characterization of layer-by-layer device structure: (a) pictures, (b) Cross section scanning electron microscopy (SEM) images of the 1.0M (left) and 1.4M (right) devices, and (c) transmittance and reflectance, (d) absorptance, (e) absorption coefficient, and (f) photon penetration length for the constituent materials/layers, as indicated. In (g) there are pictures of the samples. The thickness summary can be found in **Table S2**.

S1.3. Impedance spectroscopy at open-circuit

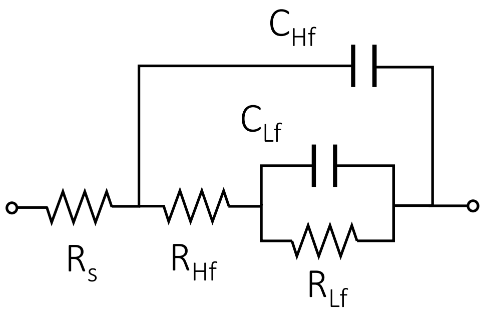

Figure S4. Equivalent circuit model[3-4, 8] used for fitting of experimental impedance spectra. Here R_s is a series resistor, R_{Hf} and R_{Lf} stand for high- and low-frequency resistors, respectively, and C_{Hf} and C_{Lf} are the high- and low-frequency capacitors, respectively.

S1.3.1. White-LED spectra

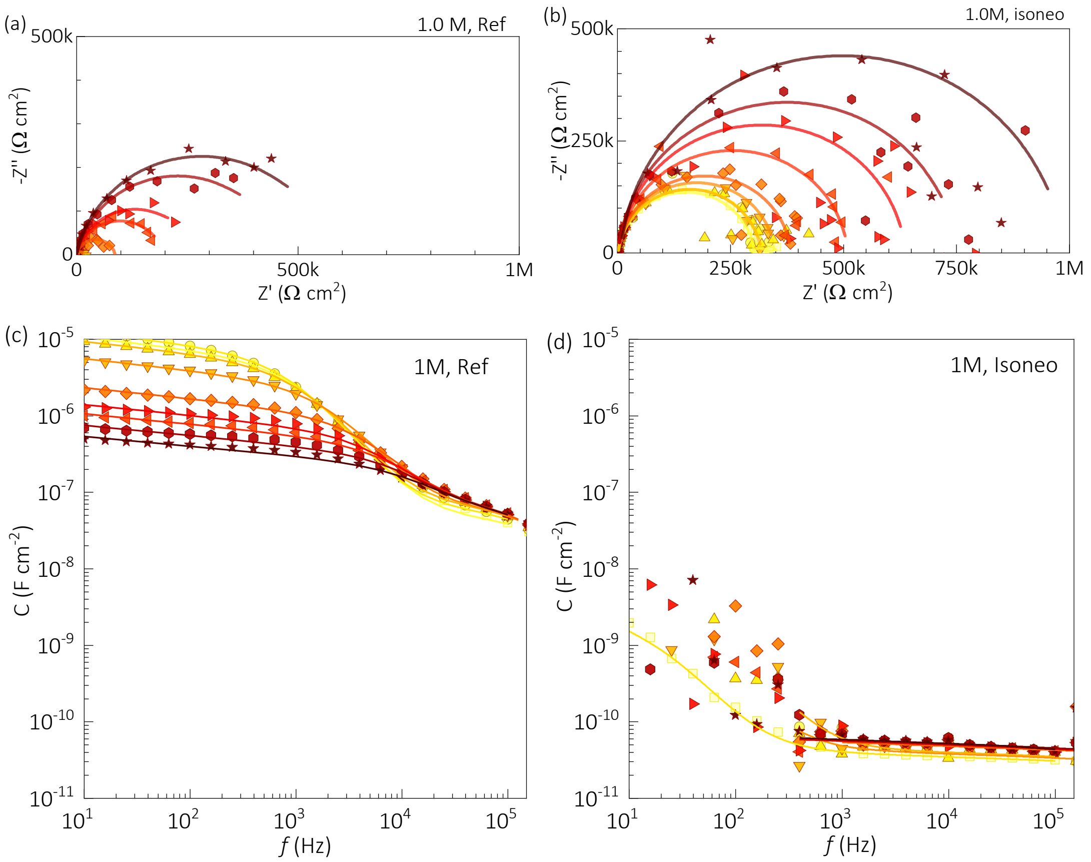

Figure S5. Impedance spectra for the 1.0 M sample in quasi-open-circuit condition under different white LED illumination intensities (a, c) without and (b, d) with passivation treatment. The spectra are illustrated in (a, b) impedance Nyquist and (c, d) capacitance Bode representations. In each case, the dots indicate experimental data, the lines are the EC fittings (see **Figure S4**), and darker and lighter colors indicate low and high irradiance intensities (and V_{oc} values), respectively.

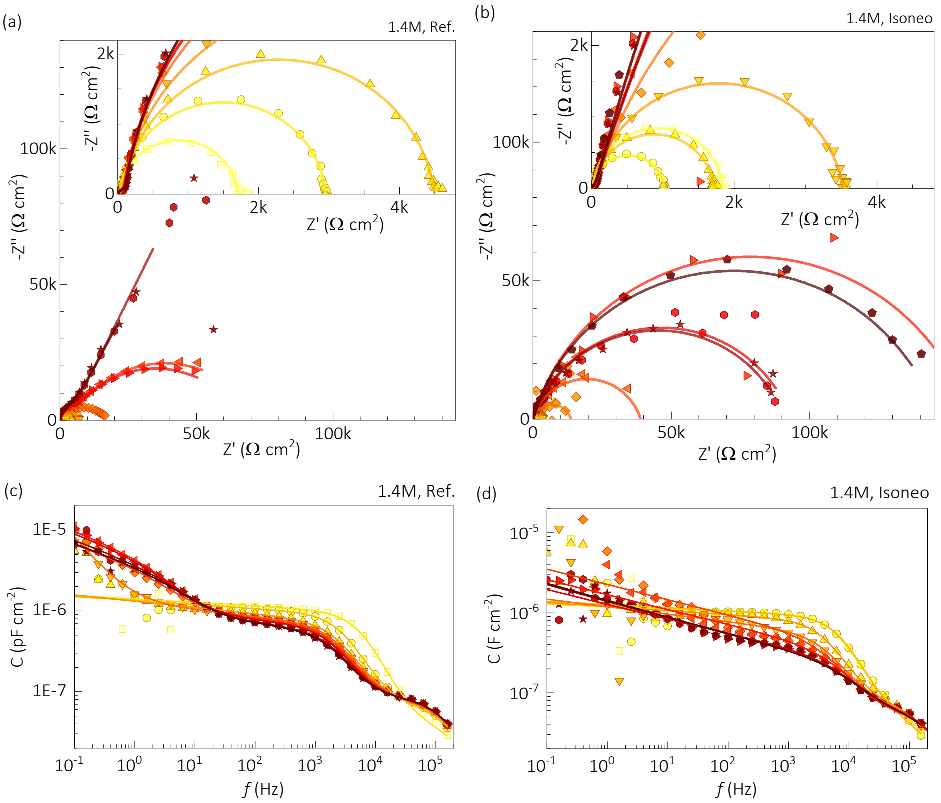

Figure S6. Impedance spectra for the 1.4 M sample in quasi-open-circuit condition under different white LED illumination intensities (a, c) without and (b, d) with passivation treatment. The spectra are illustrated in (a, b) impedance Nyquist and (c, d) capacitance Bode representations. In each case, the dots indicate experimental data, the lines are the EC fittings (see **Figure S4**), and darker and lighter colors indicate low and high irradiance intensities (and V_{oc} values), respectively.

S1.3.2. UV 365 nm-LED spectra

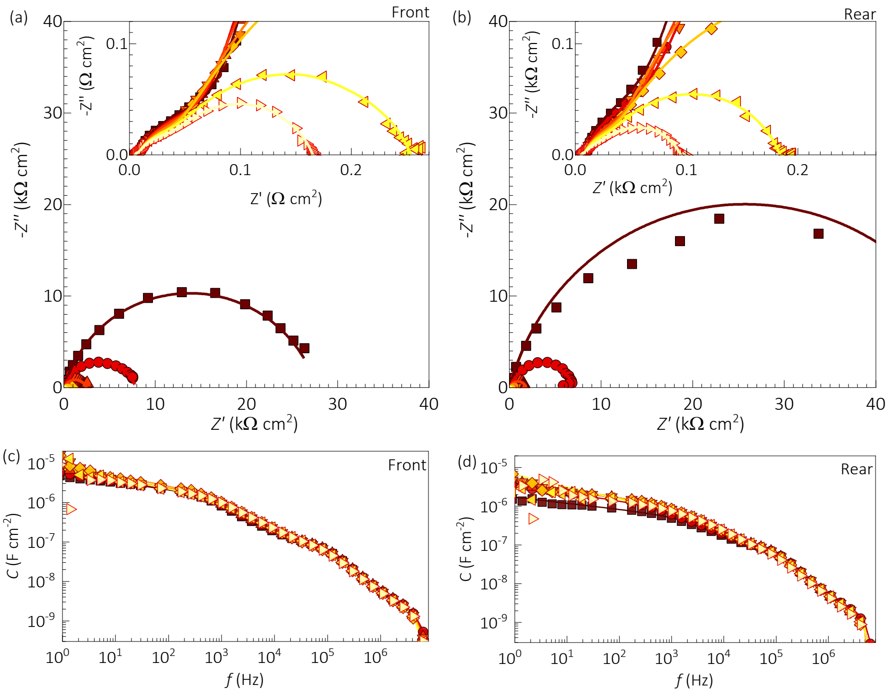

Figure S7. Impedance spectra for the 1.0 M isoneo sample in quasi-open-circuit condition under different UV irradiance intensities (365 nm LED) in (a, c) front and (b, d) rear directions of irradiance. The spectra are illustrated in (a, b) impedance Nyquist and (c, d) capacitance Bode representations. In each case, the

dots indicate experimental data, the lines are the EC fittings (see **Figure S4**), and darker and lighter colors indicate low and high irradiance intensities (and V_{oc} values), respectively.

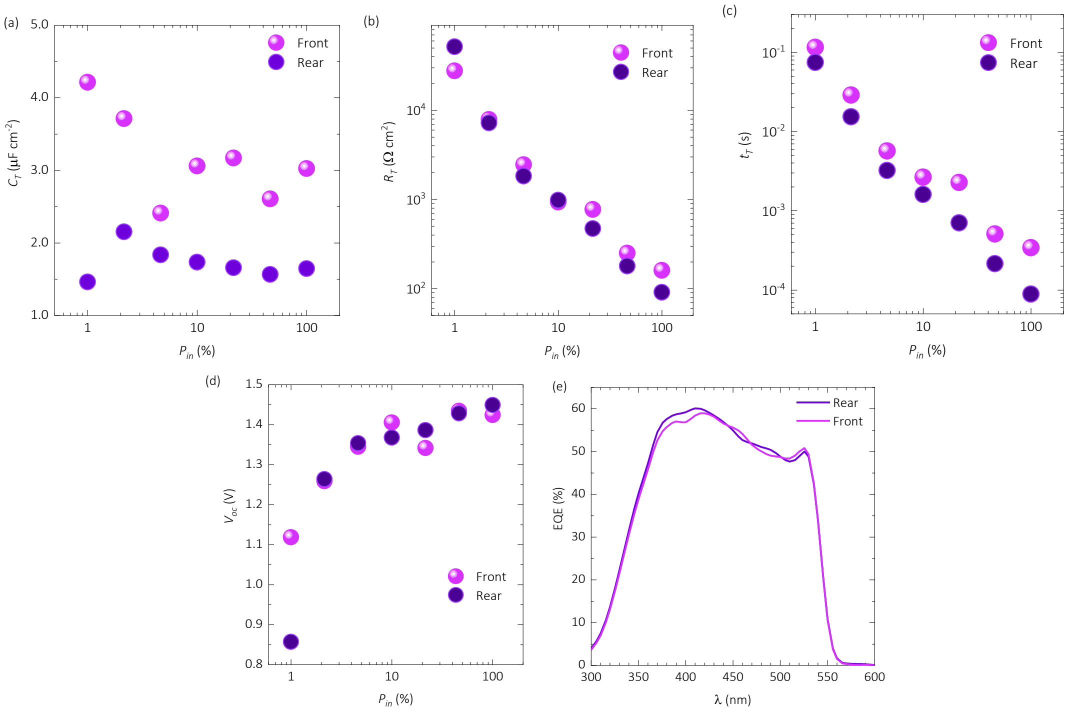

Figure S8. Impedance spectroscopy parameterization of spectra in **Figure S7** (1.0M) following equivalent circuit model of **Figure S4**, for different DC illumination intensities of the 365 nm LED. The results for front and rear directions of irradiance are shown for the summation of (a) capacitance, (b) resistance, and (c) characteristic response time. In (d) the open-circuit voltage is also presented and in (e) the EQE spectra.

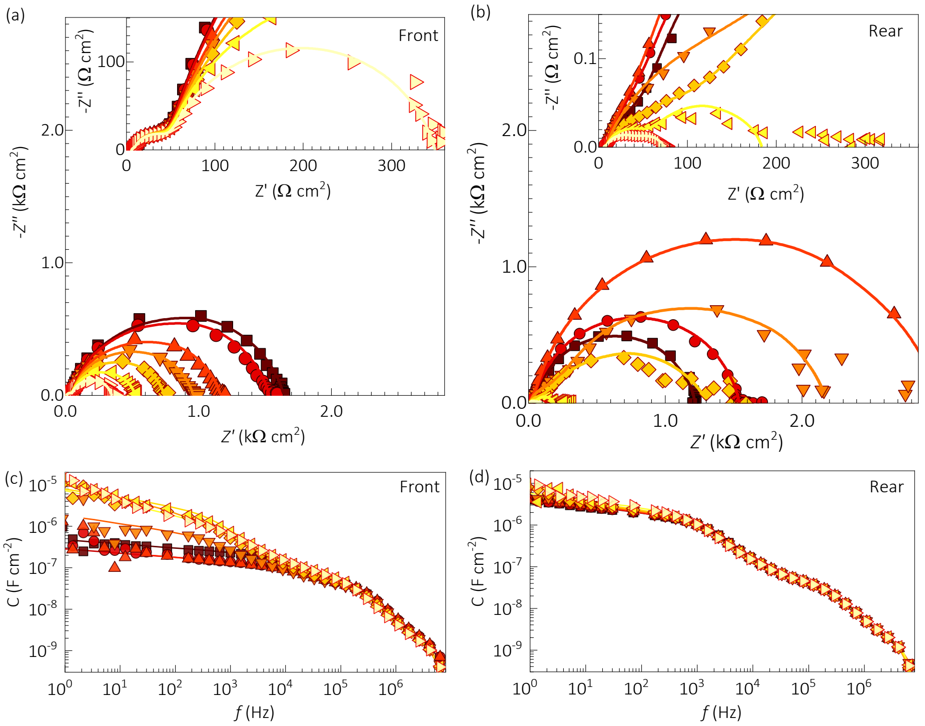

Figure S9. Impedance spectra for the 1.4 M isoneo sample in quasi-open-circuit condition under different UV irradiance intensities (365 nm LED) in (a, c) front and (b, d) rear directions of irradiance. The spectra are illustrated in (a, b) impedance Nyquist and (c, d) capacitance Bode representations. In each case, the dots indicate experimental data, the lines are the EC fittings (see **Figure S4**), and darker and lighter colors indicate low and high irradiance intensities (and V_{oc} values), respectively.

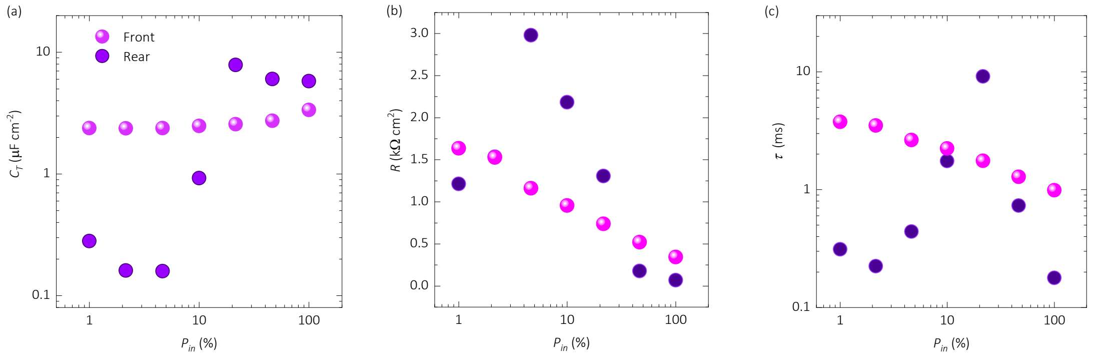

Figure 10. Impedance spectroscopy parameterization of spectra in **Figure S9** (1.4 M) following equivalent circuit model of **Figure S4**, for different DC illumination intensities of the 365 nm LED. The results for front and rear directions of irradiance are shown for the summation of (a) capacitance, (b) resistance, and (c) characteristic response time.

S2. Drift-diffusion numerical simulations

Table S3. Simulation parameters for Setfos Fluxim.[5]

**Parameter**	**Unit**	**FTO**	**TiO_2**	**$FAPbBr_3$**	**PTAA**	**ITO**
Dielectric constant	a.u.	—	20	24	3.5	—
Thickness	nm	220	30	150[a]; 255[b]	40	220
Electron affinity	eV	4.25	-4.00	-3.80	-2.70	- 5.20
Ionization potential	eV	—	-7.20	-6.10	-5.50	—
Bandgap energy	eV	—	3.20	2.3	2.80	—
Bimolecular recombination pre-factor	$cm^3 s^{-1}$	—	0	1.00e-09	0	—
Trap density	cm^3	—	0	1.00e16[a]; 5.00e16[b]	0	—
Electron pseudo-lifetime	s	—	0	5.00e-11[a]; 1.00e-11[b]	0	—
Holes pseudo-lifetime	s	—	0	1.00e-9[a]; 2.00e-10[b]	0	—
Electron capture rate	cm^3s^{-1}	—	0	2.00e-06	0	—
Hole capture rate	cm^3s^{-1}	—	0	1.00e-07	0	—
Electron diffusion length	nm	—	0	49[a]; 22[b]	0	—
Hole diffusion length	nm	—	0	222[a]; 99[b]	0	—
Electron mobility	$cm^2V^{-1}s^{-1}$	—	5.14e-4	19	1e-10	—
Hole mobility	$cm^2V^{-1}s^{-1}$	—	1e-8	19	1e-4	—
Mobile ion mobility	$cm^2V^{-1}s^{-1}$	—	—	1.00e-10	—	—
Mobile ion concentration	cm^{-3}	—	—	1e19	—	—
Concentration of acceptors	cm^{-3}	—	0	0	1e35	—
Concentration of donors	cm^{-3}	—	1e31	0	0	—
Effective density of states at EC/LUMO	cm^{-3}	—	1e33	1.00e19	1e32	—
Effective density of states at EC/LUMO	cm^{-3}	—	1e33	1.00e19	1e32	—

[a] sample 1.0M; [b] sample 1.4M

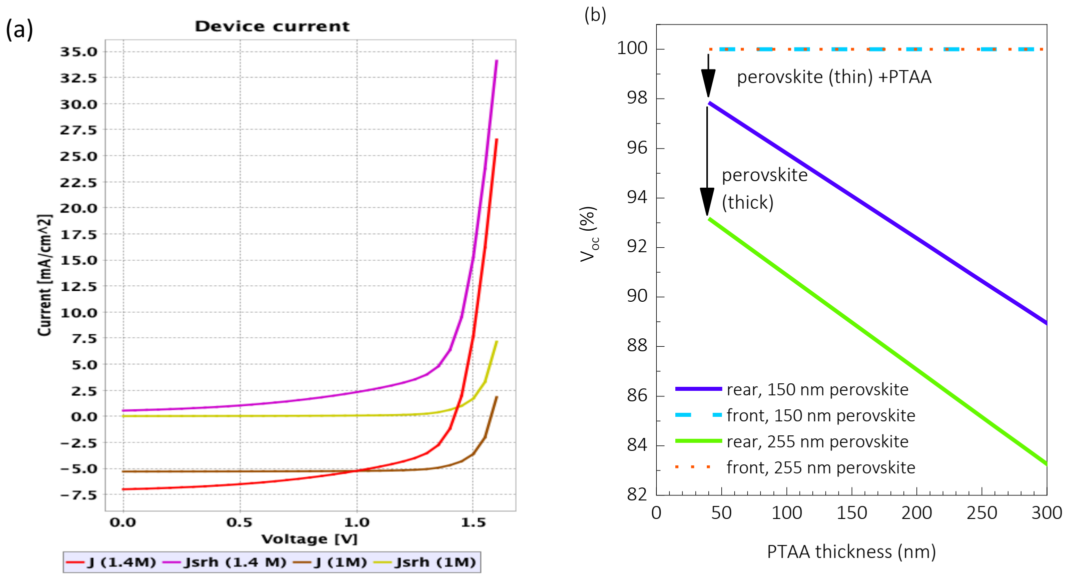

Figure S11. Drift-diffusion simulations of the effect of perovskite thickness modification including thinner (1.0M, 150 nm) and thicker (1.4M, 255 nm) samples: (a) current-voltage curves in dark and under 1 sun illumination intensity, and (b) open circuit voltage as a function of the thickness of the PTAA layer under 365 nm UV irradiance, considering the parameters in **Table S3**. In (b), the photovoltage values are normalized with respect to the respective values in front direction of irradiance.

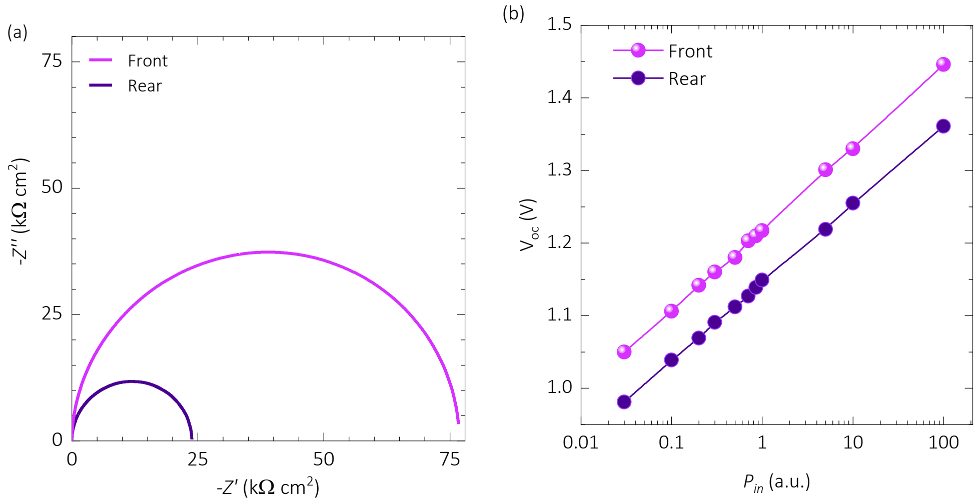

Figure S12. Drift-diffusion simulations of the effect of direction of UV irradiance (365 nm-photons): (a) impedance Nyquist plots in quasi-open-circuit conditions at the same irradiance intensity, and (b) open-circuit voltage for different incident power densities of UV irradiance. The simulation parameters are in **Table S3**.

AUTHOR INFORMATION

Corresponding authors

Osbel Almora — *Department of Electronic, Electric and Automatic Engineering, Universitat Rovira i Virgili, Tarragona 43007, Spain*; https://orcid.org/0000-0002-2523-0203 ; Email: osbel.almora@urv.cat

Fabio Matteocci — *Center for Hybrid and Organic Solar Energy, Department of Electronics Engineering, University of Rome «Tor Vergata», Via del Politecnico 1, Roma 00133, Italy; https://orcid.org/0000-0001-7893-1356* ; Email: *fabio.matteocci@uniroma2.it*

Juan Antonio Anta — *Center for Nanoscience and Sustainable Technologies (CNATS), and Department of Physical, Chemical and Natural Systems, Universidad Pablo de Olavide, Sevilla 41013, Spain; https://orcid.org/0000-0002-8002-0313*; Email: *anta@upo.es*

Authors

Farshad Jafarzadeh — *Center for Hybrid and Organic Solar Energy, Department of Electronics Engineering, University of Rome «Tor Vergata», Via del Politecnico 1, Roma 00133, Italy;* *https://orcid.org/0000-0002-6722-9075*

Mohamed Samir — *Department of Electronic, Electric and Automatic Engineering, Universitat Rovira i Virgili, Tarragona 43007, Spain;* *https://orcid.org/0000-0002-9827-1268*

Renán Escalante — *Center for Nanoscience and Sustainable Technologies (CNATS), and Department of Physical, Chemical and Natural Systems, Universidad Pablo de Olavide, Sevilla 41013, Spain;* *https://orcid.org/0000-0002-5100-5448*

Diego Di Girolamo — *Center for Hybrid and Organic Solar Energy, Department of Electronics Engineering, University of Rome «Tor Vergata», Via del Politecnico 1, Roma 00133, Italy;* *https://orcid.org/0000-0001-6307-1138*

Jessica Barichello — *Center for Hybrid and Organic Solar Energy, Department of Electronics Engineering, University of Rome «Tor Vergata», Via del Politecnico 1, Roma 00133, Italy;* *https://orcid.org/0000-0003-1277-4217*

Francesca Brunetti — *Center for Hybrid and Organic Solar Energy, Department of Electronics Engineering, University of Rome «Tor Vergata», Via del Politecnico 1, Roma 00133, Italy;* *https://orcid.org/0000-0003-2287-4545*

Lluis F. Marsal — *Department of Electronic, Electric and Automatic Engineering, Universitat Rovira i Virgili, Tarragona 43007, Spain;* *https://orcid.org/0000-0002-5976-1408*

Notes: The authors declare no competing financial interest.

Acknowledgments: J.A.A acknowledges the Ministerio de Ciencia e Innovación of Spain, Agencia Estatal de Investigación (AEI) and EU (FEDER) under grants TED2021-129758B-C33 (TransEL), PID2022-140061OB-I00 (DEEPMATSOLAR), and PCI2019-111839-2 (SCALEUP). O.A. acknowledges the Juan de la Cierva Fellowship grant FJC2021-046887-I funded by MICIU/AEI/ 10.13039/501100011033 and by the European Union NextGenerationEU/PRTR.

References

[1] D. Di Girolamo, G. Vidon, J. Barichello, F. Di Giacomo, F. Jafarzadeh, B. Paci, A. Generosi, M. Kim, L.A. Castriotta, M. Frégnaux, J.-F. Guillemoles, F. Brunetti, P. Schulz, D. Ory, S. Cacovich, A. Di Carlo and F. Matteocci, Breaking 1.7 V Open Circuit Voltage in Large Area Transparent Perovskite Solar Cells Using Interfaces Passivation, *Adv. Energy Mater.* **2024**, 2400663. https://doi.org/10.1002/aenm.202400663

[2] M.V. Khenkin, E.A. Katz, A. Abate, G. Bardizza, J.J. Berry, C. Brabec, F. Brunetti, V. Bulović, Q. Burlingame, A. Di Carlo, R. Cheacharoen, Y.-B. Cheng, A. Colsmann, S. Cros, K. Domanski, M. Dusza, C.J. Fell, S.R. Forrest, Y. Galagan, D. Di Girolamo, M. Grätzel, A. Hagfeldt, E. von Hauff, H. Hoppe, J. Kettle, H. Köbler, M.S. Leite, S. Liu, Y.-L. Loo, J.M. Luther, C.-Q. Ma, M. Madsen, M. Manceau, M. Matheron, M. McGehee, R. Meitzner, M.K. Nazeeruddin, A.F. Nogueira, Ç. Odabaşı, A. Osherov, N.-G. Park, M.O. Reese, F. De Rossi, M. Saliba, U.S. Schubert, H.J. Snaith, S.D. Stranks, W. Tress, P.A. Troshin, V. Turkovic, S. Veenstra, I. Visoly-Fisher, A. Walsh, T. Watson, H. Xie, R. Yıldırım, S.M. Zakeeruddin, K. Zhu and M. Lira-Cantu, Consensus statement for stability assessment and reporting for perovskite photovoltaics based on ISOS procedures, *Nat. Energy* **2020**, *5*, 35-49. https://doi.org/10.1038/s41560-019-0529-5

[3] E. Ghahremanirad, O. Almora, S. Suresh, A.A. Drew, T.H. Chowdhury and A. R. Uhl, Beyond Protocols: Understanding the Electrical Behavior of Perovskite Solar Cells by Impedance Spectroscopy, *Adv. Energy Mater.* **2023**, *13*, 2204370. https://doi.org/10.1002/aenm.202204370

[4] O. Almora, Y. Zhao, X. Du, T. Heumueller, G.J. Matt, G. Garcia-Belmonte and C.J. Brabec, Light Intensity Modulated Impedance Spectroscopy (LIMIS) in All-Solid-State Solar Cells at Open-Circuit, *Nano Energy* **2020**, *75*, 104982. https://doi.org/10.1016/j.nanoen.2020.104982

[5] SETFOS: Simulation Software for Organic and Perovskite Solar Cells and LEDs, https://www.fluxim.com/setfos-intro (accessed 28.03.2024)

[6] C. Yang, D. Liu, M. Bates, M.C. Barr and R.R. Lunt, How to Accurately Report Transparent Solar Cells, *Joule* **2019**, *3*, 1803-1809. https://doi.org/10.1016/j.joule.2019.06.005

[7] O. Almora, C.I. Cabrera, J. Garcia-Cerrillo, T. Kirchartz, U. Rau and C.J. Brabec, Quantifying the Absorption Onset in the Quantum Efficiency of Emerging Photovoltaic Devices, *Adv. Energy Mater.* **2021**, *11*, 2100022. https://doi.org/10.1002/aenm.202100022

[8] O. Almora, K.T. Cho, S. Aghazada, I. Zimmermann, G.J. Matt, C.J. Brabec, M.K. Nazeeruddin and G. Garcia-Belmonte, Discerning Recombination Mechanisms and Ideality Factors through Impedance Analysis of High-Efficiency Perovskite Solar Cells, *Nano Energy* **2018**, *48*, 63-72. https://doi.org/10.1016/j.nanoen.2018.03.042
